# *TP53* in Acute Myeloid Leukemia: Molecular Aspects and Patterns of Mutation

**DOI:** 10.3390/ijms221910782

**Published:** 2021-10-05

**Authors:** Binsah George, Hagop Kantarjian, Natalia Baran, Joseph Douglas Krocker, Adan Rios

**Affiliations:** 1 Department of Hematology and Oncology, University of Texas Health Science-Houston, Houston, TX 77030, USA; joseph.krocker@uth.tmc.edu (J.D.K.); Adan.rios@uth.tmc.edu (A.R.); 2 Department of Leukemia, The University of Texas—MD Anderson Cancer Center-Houston, Houston, TX 77030, USA; hkantarjan@mdanderson.org (H.K.); nbaran@mdanderson.org (N.B.)

**Keywords:** *TP53* mutations, acute myeloid leukemia, clonal hematopoiesis, variable allele frequency

## Abstract

Mutation of the tumor suppressor gene, *TP53*, is associated with abysmal survival outcomes in acute myeloid leukemia (AML). Although it is the most commonly mutated gene in cancer, its occurrence is observed in only 5–10% of de novo AML, and in 30% of therapy related AML (t-AML). *TP53* mutation serves as a prognostic marker of poor response to standard-of-care chemotherapy, particularly in t-AML and AML with complex cytogenetics. In light of a poor response to traditional chemotherapy and only a modest improvement in outcome with hypomethylation-based interventions, allogenic stem cell transplant is routinely recommended in these cases, albeit with a response that is often short lived. Despite being frequently mutated across the cancer spectrum, progress and enthusiasm for the development of p53 targeted therapeutic interventions is lacking and to date there is no approved drug that mitigates the effects of *TP53* mutation. There is a mounting body of evidence indicating that p53 mutants differ in functionality and form from typical AML cases and subsequently display inconsistent responses to therapy at the cellular level. Understanding this pathobiological activity is imperative to the development of effective therapeutic strategies. This review aims to provide a comprehensive understanding of the effects of *TP53* on the hematopoietic system, to describe its varying degree of functionality in tumor suppression, and to illustrate the need for the adoption of personalized therapeutic strategies to target distinct classes of the p53 mutation in AML management.

## 1. Introduction

The *TP53* gene and its protein was first described in 1979 and has since taken center stage due to an avalanche of research after mutations in this gene were identified as the common denominator in more than 50% of human cancers. *TP53* mutation associated acute myeloid leukemia represents a distinct molecular cohort with a notorious reputation for uniformly poor prognosis [1]. The *TP53* mutation confers a resistant predisposition to current therapeutics regardless of age. In comparison, CR in a non-*TP53* mutated cohort is around 80% [2,3,4,5]. A lower variant allele frequency (VAF) of the *TP53* mutation is associated with fewer chromosomal losses and a complex karyotype but does not confer a better overall or event free survival to a higher allele frequency [6].

This poor outcome could be substantiated by the crucial role p53 imparts in mediating apoptotic feedback to standard chemotherapy leading to inherent resistance in mutated p53 [2,5,7,8,9].

Li-Fraumeni syndrome, a condition caused by a germline mutation in *TP53* in humans born with a single mutant allele of *TP53*, encompasses a wide variety of early onset cancers, including AML in around 5% of Li Fraumeni related malignancies [10].

Somatic mutations in transcription factor *TP53* represent one of the most frequently mutated alterations in human cancers. These mutations are surprisingly observed in only 5–10% of de novo AML but occur in about 25% of older patients with de novo AML with a median age of 60–67 years [3]. In genomic, unstable AML, like t-AML therapy related myelodysplastic syndrome(t-MDS), *TP53* mutation is observed in 30–35% of cases. However, in complex-karyotype AML *TP53* is mutated in 70% of proportion. Therefore, TP53 represents a powerful adverse indicator of poor prognosis independent of complex karyotype, age and other genetic markers [11,12,13,14,15].

Broadly, *TP53* is mutated in all morphological subtypes of the French American British (FAB) classification but is more enriched in erythroleukemia (25–36%) [16,17].

The relative lower prevalence of somatic *TP53* mutation in AML compared to solid tumors like ovarian (48%), and colorectal (43%) suggests that other cooperating events are necessary for leukemic progression [3].

## 2. *TP*53 Gene, p53 Structure and Cellular Functions

**Figure 1:**
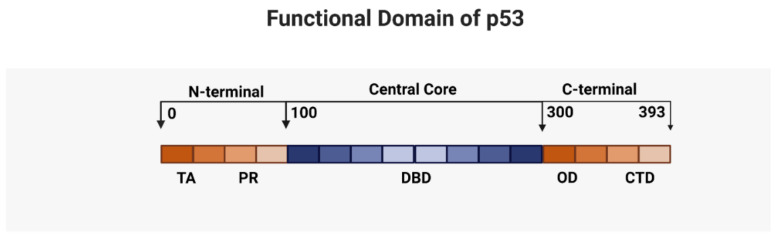
Functional domain of p53: The N-terminal portion consists of the transactivation domain (TA) and the proline rich domain (PR). TA is required for transactivation of various transcription factors and interaction of MDM2 ubiquitin ligase. The central core is mainly made of the DNA—binding domain (DBD); most of the exons of *TP53* are sequenced. The C—terminal consists of the oligomerization domain (OD) and the carboxy- terminal regulatory domain.

Normal (wild type, WT) p53 protein, which is encoded by the *TP53* gene, is located on chromosome 17p13.1. The p53 protein product is a 393 amino acid long phosphoprotein which contains five important hallmark domains: the amino N-terminal transactivation domain, a central DNA-binding domain (DBD), a carboxy-terminal oligomerization domain, and a regulatory domain. (Figure 1) [18]. P53 is known as the guardian of the genome as its official function is to maintain harmony between cell arrest and cell growth during genomic stress [19].

*TP53* gene is largely a stress response protein with functions ranging from apoptosis to cell cycle control. Therefore, a mutation in the *TP53* gene pronounces the effects of oncogenes leading to the uncontrolled proliferation of tumor cells. These mutations are known to give rise to a deleterious gain of function (GOF), a loss of function (LOF) or a non-mutational dysfunction or inactivation of the p53 protein [20,21,22,23]. There are multiple pathways to p53 inactivation resulting in decreased p53 level and subsequent cell proliferation. Half of the time this inactivation is a direct result of *TP53* gene mutation in one *TP53* allele which eventually can lead to the loss or partial inactivation of the other WT allele over time or during disease progression, owing to loss of heterozygosity (LOH). LOH, in the context of somatic and germline mutation, is considered as a gene alteration that further evolves to tumor progression [24,25,26].

p53 is normally ‘off’ with a short half-life and is tidily regulated at the protein level via post-translational modifications including ubiquitination, phosphorylation, acetylation and methylation [27,28,29,30]. It is activated when cells are stressed [31] or damaged avoiding further proliferation of stressed cells through the G1/S phase of the cell cycle. The continuous ubiquitylation and subsequent degradation allow the unstressed cell to maintain a low level of WT p53. In contrast, p53 ubiquitylation is suppressed under hypoxic conditions, or oncogene activation and DNA damage, leading to its accumulation. p53 is then stabilized in the nucleus in the form of tetrameric complexes and finally undergoes its activation. The formation of tetramers is required for p53 to be fully active and serve as a transcriptional activator of more than 150 target genes [26,32].

The fate of p53 protein in the cell is mainly determined by the rate of degradation rather than the rate of production. p53 is primarily degraded by the interaction of p53 with MDM2 (Double minute 2 Protein).

MDM2 is a ubiquitin E3 ligase that labels p53 with ubiquitin, resulting in p53 degradation by 26S proteasomes [33]. This interaction of p53/MDM2 can be disrupted when stress or DNA damage is detected resulting in the stabilization of p53. Of note, an overexpression of MDM2, which can be observed in many tumors, leads to the deactivation of p53 [26].

Interestingly, primary AML with WT *TP53* can overexpress high levels of another important regulator of p53 called MDM4 (also known as MDMX), through binding of p53 AT domain. This finding demonstrates the detrimental consequences of dysfunctional p53 pathways accompanied by inferior survival.

To the contrary, mutant p53 accumulates in tumor cells and is regulated and stabilized by MDM2 in a manner similar to WT p53, which could be secondary to attainting a (GOF) phenotype. Therefore, detection of increased levels of p53 by immunohistochemistry (IHC) correlates with *TP53* gene mutation as p53 expression is low in a normal cell [27,28,29,30].

The p53 network is activated primarily by three mechanisms which maintain an increased concentration of p53 protein. This stabilization of higher concentrations of p53 allows for p53 binding to DNA sequences with ensuing transcription of adjacent genes, ultimately resulting in inhibition of cell division or cell death. The first pathway is the activation of kinases like ataxia telangiectasia related (ATR) and casein kinase II on exposure to chemotherapeutic agents and ultraviolet light [34]. Secondly, an increased oncogene expression such as that of Myc or Ras triggers growth signals resulting in the production of p14ARF protein which consequently activates p53 by binding to MDM2 and inhibiting its activity [35,36,37]. Lastly, major kinases like ATM (ataxia telangiectasia mutated) stimulate Chk2 (Checkpoint kinase 2) when triggered by double strand breaks [37]. In summary, significant DNA dependent protein kinases including ATM, Chk1, and Chk2 sense DNA damage, phosphorylate p53 at the AT (amino terminal domain) sites close to the MDM2 binding region and consequently block MDM2 interaction with p53. The end result of this cascade is the stabilization of p53. [38].

p53 encompasses around 18,000 mutations in different cancers. Unlike the majority of the tumor suppressor variants which largely consist of truncation mutations, the predominant p53 mutation in leukemia is a monoallelic missense mutation [39,40]. A missense mutation is a point mutation in the DNA resulting in a single amino acid substitution within the translated protein. This mutation produces a phenotype with increased expression of altered p53 protein, which accumulates in tumor cells contributing to tumor initiation, promotion and chemoresistance [38]. The alternative mutations such as nonsense, splice-site or insertion/deletions are observed in lower frequencies [39,41].

Monoallelic mutation gives rise to mutant p53 full-length protein which are detected in tumors and usually mapped in the DBD affecting sequence specific DNA-binding activity [24].

*TP53* mutation in leukemia can occur in the context of somatic and germline mutations, the latter of which is associated with Li-Fraumeni syndrome leads to the development of specific solid tumors and is less likely to arise in diverse tissues [41]. In the sporadic setting, however, *TP53* mutations seem to occur when exposed to carcinogens (environmental factors), oncogenic agents or genotoxic insults. This observation further elucidates the robust functionality of p53 in maintaining genomic stability and preventing tumor formation [3].

## 3. Role of *TP53* in Hematopoiesis, Clonal Hematopoiesis and AML

During the development of an organism, each cell acquires a similar set of genes and a template genetic sequence. Differentiation of cells into specific tissues and their function is a byproduct of the activation or silencing of certain genes. With age or as a result of exposure to environmental mutagens, proliferating and differentiating cells may procure somatic mutations. The effects of mutation are many including neutral effects if the variant lies within a non-regulatory region, an increase in proliferation capacity or negative selection against cells if contained within essential genes [42,43].

The hematopoietic system is a complex yet efficient entity responsible for producing a multitude of cells daily. To meet these demands, hematopoietic stem cells (HSCs) have a hierarchical methodology of self-renewal and division into progenitor cells. HSCs can divide giving rise to a progenitor and another HSC or two progenitor cells which will later divide and differentiate into mature cells. Progenitor cells have a limited self-renewal potential. In these cells the acquisition of a mutation will usually end the clonal lineage. However, in some cases a mutation can have a proliferative advantage leading to progenitor cell proliferation. Occasionally, the slowly dividing HSCs may acquire mutations which can influence the HSC compartment and daughter cells. These variants may lead to proliferation and expansion of the HSC clone or one lineage of cells. This constitutes the basis of clonal hematopoiesis (CH) [44,45,46,47].

An important role in the self-renewal and proliferation of normal HSCs is assigned to the TP53 gene and p53 protein [48]. Under biologically functional *TP53* HSC self-renewal is decreased when exposed to DNA damaging radiation, oncogene activation and alkylating agents. However, *TP53* disruption leads to augmented proliferation and overproduction of pluripotent stem cells [49,50,51]. This suggests that suppression of p53, or its pathway, leads to outgrowth of pluripotent stem cells with subsequent malignant transformation of hematopoietic stem/progenitor cells [52,53,54].

Early studies have shown p53 to be involved in apoptosis, proliferation and differentiation of human HSCs and provides quiescence during steady states of hematopoiesis [55,56]. Intact p53 is integral to the function of bone marrow mesenchymal stromal cells (MSC). This is evidenced by observations that mice with p53 deficient MSCs were unable to support hematopoietic progenitors [57]. Functional p53 is known to contribute to HSC genetic stability and homeostasis by reducing intracellular reactive oxygen species level [58]. In murine HSCs with p53 loss and incorporation of mutant KrasG12D, there was a disposition for indefinite self-renewal and the ability to transform into leukemia cells [51].

With modern techniques, it is now widely understood that aging is ubiquitously associated with mutations and HSC expansion that can harbor mutations in leukemia specific genes without associated hematologic malignancies or cytopenia, commonly referred to as age related clonal hematopoiesis (ARCH) [59,60].

Clonal hematopoiesis of indeterminate potential (CHIP) in leukemia associated genes including DNMT3A, ASXL1, TET2 with a variant allele frequency (VAF) of ≥2% can confer a predisposition for hematological malignancy including overt leukemia, as well as thrombosis, stroke and cardiovascular events [61,62,63,64]. More recently, the utilization of targeted deep sequencing techniques has allowed for the identification of VAFs of >0.5–1%, which have been noted to be associated with an increased odds of AML development [60,63,65]. For this review we will refer to ARCH and CHIP as clonal hematopoiesis (CH)

*TP53* somatic mutation is associated with aging, is present in 4% of CH and is believed to play a vital role in CH development along with other associated gene mutations in DNMT3A, TET2, ASXL1, SRSF2, CBL, and SF3B1. There is strong evidence that when the *TP53* mutation is present it represents a nearly initiating event and presents as a dominant or founding clone in the development of leukemia whereas DNMT3A and TET2 mutations are sustained over time with no overt association with AML development [11,66].

*TP53* mutations are associated with CH development and there is convincing evidence that mutated *TP53* can lead to a permissive state of overt leukemia. These findings support the perceived fitness advantage conferred by *TP53* through enhancement of HSC self-renewal in light of genotoxic stresses [47]. However, Trp53 mutation induced mouse models exhibit no overt transformation to leukemia, enforcing the idea that Trp53 mutation alone is not sufficient for a foolproof leukemia initiation, but rather multiple additional steps are required [51,67].

Serial acquisition of somatic mutations is known to be involved in the pathogenesis of AML; nevertheless, more definitive studies are needed to address whether these mutations are an initiating or cooperating event. This continues to be a challenge as one third of AML patients harbor no mutations in known leukemia associated genes [68]. Hence, despite the understanding of CH having an effect on survival, there are currently no data supporting the screening of asymptomatic patients.

## 4. Functional Patterns of *TP53* Mutations

In clinical settings, all *TP53* mutations are viewed collectively, and therapeutic intervention is not stratified by specific TP53 genotype. However, a plethora of research has enabled us to understand various functional subgroups within p53. Understanding the influence of various forms of *TP53* mutations on the clinical characteristics of leukemia is useful in grasping the underlying p53 biology and critical for the development of potent targeted agents [69].

The changes in gene and subsequent protein functionality can be studied based on mutation site in reference to the domain-based structure of p53 protein.

The three main functional regions of p53 protein are the amino terminal region, the centrally situated DNA-binding domain and the oligomerization domain [70]. In AML, *TP53* mutations were detected most frequently in the centrally situated DNA-binding domain, followed by the amino terminal domain and the oligomerization domain [70]. The different *TP53* domains are distinct in functional activity and under normal circumstances all p53 mutants are inert and behave as LOF mutants. However, exposure to genotoxic stress can have varied effects including GOF and dominant negative (DN) effects (Figure 1) [71,72]. The characterization of the three main functional domains is presented below.

### 4.1. Amino Terminal Domain Mutants (AT)

The AT domain of the *TP53* gene spans through amino acid residues 1-61 and encodes for a transcriptional activator which harbors the transactivation domains (TAD) 1 and 2 and a proline rich domain. Although, the explicit molecular function of TAD1 and TAD2 remains obscure, TAD1 is associated with the induction of a number of genes while TAD2 contributes to tumor suppression. Both TAD1 and TAD2 can independently transactivate genes, however, at least one TAD is required for p53 transcription [73].

AT mutations within the first 40 amino acids are usually insertion or deletion variants and result in the abrogation of full-length p53 expression [74]. The AT domain has several significant regulatory regions, including MDM2. This oncoprotein is a cellular inhibitor of p53 that can bind to the AT domain causing p53 degradation via ubiquitylation and p300 binding resulting in p53 deactivation via acetylation. Some cancers can lead to the amplification of MDM2 resulting in p53 inactivation and unopposed cell growth [75,76].

When a full length p53 formation is disrupted from mutations in the AT domain, initiation of translation from an alternative codon expresses p47. This isoform of p53 is a truncated 47 kDa protein and, when endogenously expressed, serves as a tumor suppressor when p53 lacks TAD1 and TAD2 [77,78].

P47 can induce the expression of selective apoptotic genes and retains some transactivation properties but is unable to encode for cell-cycle arrest genes such as CDKN1A [79,80]. P47 retains a selective apoptotic function despite a flawed G1/S cell cycle checkpoint. Nevertheless, AT domain mutations are prognostically more favorable than p53 DBD mutations.

Hence, expression of high p47 or AT domain *TP53* mutations have a better response to therapy which translates into improved overall survival [81]. Theoretically, p47 expression could be used as a biomarker to predict favorable response to therapy

### 4.2. DNA Binding Domain (DBD)

The centrally located DBD is the most frequently mutated (around 80%) region and spans amino acids 100–200 within exons 5–8 (Figure 1). Mutations to this domain result in the inability to transactivate multiple target genes leading to the functional inactivation of p53 and the loss of DNA binding capacity [71,82]. DBD carries six major mutational hot spots including codons 175, 245, 248, 249, 273, and 282, showing a propensity for arginine residues [83]. The most common hot spot DBD mutations reside in codon 175. The first 42 amino acids at the N-terminal transactivation domain interact with the basal transcriptional machinery serving as a positive regulator of gene expression [69].

As mentioned earlier and contrary to popular belief, mutations often cause various functional defects at a cellular level and not solely LOF via loss of tumor suppressor activity and mitigation of transcriptional activation of the p53 gene [84]. However, in general, DBD mutants are usually LOF variants.

As evident in LFS and mouse models, one DBD mutant *TP53* allele confers a predisposition to spontaneous cancer development [85]. The distribution of hot spot residues in germline and sporadic tumors are similar [86]. Thus, the loss or mutation of both *TP53* alleles accentuates spontaneous tumor development raising the importance of gene dosage and the enhanced oncogenic potential of the p53 missense mutation [71,87]. Generally, with an underlying DBD mutant p53 allele tumorigenesis is accentuated in the presence of DNA damaging agents. The resulting effects are similar to that of the null p53 allele (*TP53* −/−) with decreased transactivation of target genes and limited function of the WT p53 leading mainly to loss of tumor suppression, apoptosis and DNA repair [88,89]. Consequently, DBD mutations are distinguished by complete loss of transactivation potential [90,91], and are resistant to various anticancer therapies resulting in a poor prognosis [77,92,93].

Usually, missense mutations can produce full length mutant p53 which is more stable than WT and present at high levels in tumor cells [94]. These variants attain additional features that inhibit the co-expression of the remaining WT protein encoded in the second allele and are known to have DN effects that support oncogenesis and survival. Of note, DN effects are expressed by the majority of the DBD mutants [29,69,95]. The DBD-DN mutant has an inhibitory effect on the remaining WT p53 protein transgressing to a lower canonical p53 functionality in comparison to LOH of the WT allele or *TP53*+/− heterozygosity [69,71,96]. Data from multiple studies have concluded that DN effect does not generally lead to tumorigenesis with loss of p53 function. DN is not usually demonstrated at basal levels in mutant p53 protein expression but rather a higher DBD mutant p53 to WT ratio of around 3:1 is required to contribute to tumorigenesis [97,98]. Exposure to DNA damaging agents including radiation and chemotherapy can increase the DBD mutant ratio inducing DN effects by decreasing the WTp53. This implies that therapeutic interventions that decreases mutant p53 could abrogate tumorigenesis in malignancy with one DBD mutant allele [69,71,96].

Often, mutant p53 can initiate new functions independent of WT p53, conferring gain-of-function (GOF) properties that propagate progression, metastasis, drug resistance and survival. GOF capability is achieved by the binding of mutant p53 to the p63 and p73 family of tumor suppressor genes resulting in functional alterations and inhibition of these key transcription factors required for cell cycle arrest [24,88,99,100]. Furthermore, mutant p53 binding to p73 protects cells from chemotherapeutic agents [101]. Thus, GOF effects the ability of the mutant p53 to transactivate novel cell survival genes, [102] which then leads cancer cells to become dependent on mutant p53 for tumorigenesis, [103,104] while resisting cell death on exposure to DNA damaging agents [105]. DBD mutants do not usually possess GOF characteristics, but when present, are commonly seen with mutations in codon 175, 248 and 273 [69]. Hence, targeting the interaction between p53 and p63 and p73 would constitute a promising strategy, thus restoring p73 activity could re-sensitize the cancer cells to therapeutic agents [106]. The aforementioned phenotypic effects of DBD mutations are not mutually exclusive.

### 4.3. Oligomerization Domain (OD)

The OD, which spans amino acid 325–356, is essential for tetramerization of p53, an important event in tumor suppressor function. Recurrent hotspots with OD mutations are R337H, R342P, and R342. Mutation within the OD is rarely seen in sporadic cancer but commonly encountered with germline mutations [107,108].

Usually, when a mutation affects tetramerization, it is known to diminish DNA binding. Consequently, mutation confers complete or partial loss of transactivation and loss of tumor suppression, thus functionally behaving like LOF p53 proteins [107,109]. Generally, patients with OD mutations lack a response to chemotherapeutic agents as they rely on a p53 mediated cytotoxic effect [69].

In summary, the mutation of various p53 domains results in a spectrum of functional consequences at a cellular level. Mutation in the AT domain is usually associated with partial loss of transactivation potential with the ability to transactivate select genes.

**Figure 2:**
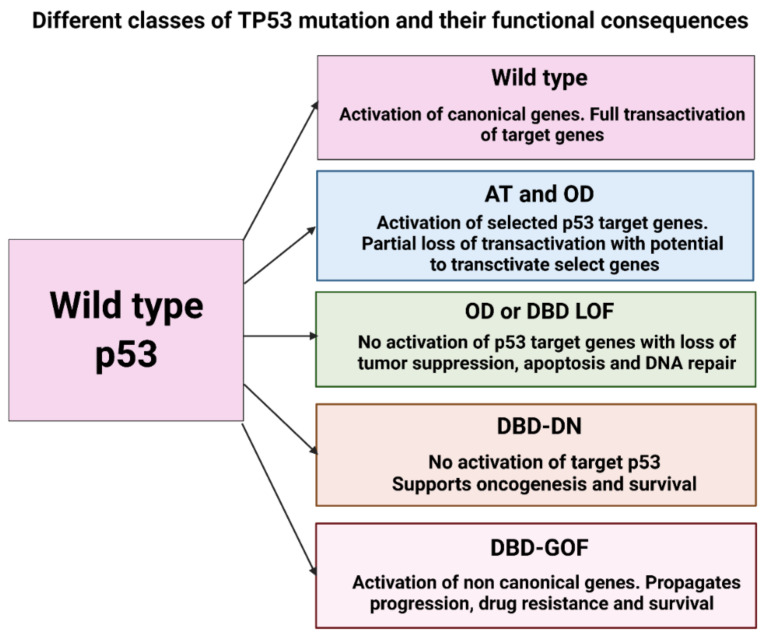
Classes of TP53 mutations and their functional implication. Amino-terminal (AT), Oligomerization domain (OD), DNA-binding Domain (DBD), Loss of function (LOF), Double negative (DN), Gain of function (GOF).

DBD-LOF variants possess the ability to deliver partial transcriptional function leading to cell death similar to OD mutants while lacking WT p53, while DBD-GOF mutants have the worst functionality with increased cellular survival and metastasis in the p53 null state. Hence, envisaging the different functional classes of the p53 mutant can drastically improve outcomes if therapeutic approaches specific to p53 mutant subtype are adopted (Figure 2) [69].

## 5. Co-occurring Mutations and Allelic States in *TP53* AML

*TP53* mutation resulting in loss or alteration of function is seen in approximately 8% of de novo AML. However, these genetic variants are present in 30% of t-AML cases and in patients with complex karyotype, especially in the elderly, they are encountered in around 70% of subjects. Nevertheless, prognosis and overall survival is poor in all subgroups [110,111,112,113]. The 2017 European Leukemic Net (ELN) guidelines was updated to include *TP53* mutated leukemia as an unfavorable group. Overall survival for patients with *TP53* mutation and complex cytogenetics (i.e., ≥3 cytogenetic abnormalities) is worse in comparison to *TP53* WT and complex karyotype or *TP53* mutated with no complex cytogenetics [66].

Presence of *TP53* mutations as part of CHIP have been observed although mutations in DNMT3A, TET2 and ASXL1 are more common compared to *TP53* [59,61,63,114]. Desai et al. evaluated the presence of mutated *TP53* in a normal population through prospective study, subjects that harbored a detectable VAF of 1% or more in *TP53* or IDH1/2 mutation eventually developed AML. RUNX1 had the shortest latency to develop AML (1.5 years vs. 9.6 years without mutation). Of note, the RUNX1 study was limited by small sample size (only 3 patients), hence more studies are needed to validate this finding. Other mutations with increased odds of developing AML included DNMT3A, TET2, and spliceosome-related genes SRSF2, SF3B1 and U2AF1 [63]. Although, a part of CH it should be noted that a VAF of ≥10% in DNMT3A and TET2 were at increased risk for AML whilst a lower VAF (<10%) was less specific to AML. However, this was not the case with *TP53* and IDH mutations, wherein the odds to develop AML was independent of VAF [63].

Mutated *TP53* and PPM1D clones were strongly enriched during and after induction chemotherapy. This finding does not necessarily represent the initiation of these mutations but is rather considered to be secondary to preferential expansion of candidate mutations that were present in small frequencies prior to cytotoxic chemotherapy [11,115].

*TP53* mutation is commonly associated with complex karyotype, chromothripsis, chromosomal arm losses (especially 5, 7, 17), cytogenetic alterations to copy number (aneuploidy) or some combination of these features [12,66,116]. Usually, *TP53* mutations are not associated with low risk aberrations like t (8:21) or inv 16 [12]. An analysis of 40 AML patients demonstrated that copy number alterations with *TP53* mutations were associated with trisomy of chromosome 8, gain of chromosome 17 (11.2), 14 (q32.3), 16p (11.2–11.3) and deletion of chromosome 12 (p12.3) [13,117]. Although the mechanism of leukemogenesis in *TP53* mutated AML is unknown, the frequent observation of chromosome 5, 7 and 17 aneuploidy with mutated *TP53* advocate for their involvement in the transition from CHIP to AML [115].

*TP53* mutations are less often linked with alterations in the RAS pathway (4%), FLT3 (6%) or NPM1 (8%), [4,112] and to a lesser frequency can co-exist with single nucleotide variants in recurrent AML genes like TET2, IDH1/2 and DNMT3A [12,13,66,112,116]. A different pattern of co-mutations may be observed within a founding clone compared to a subclone with *TP53* mutation. If associated with a founding clone, *TP53* mutation usually coexists with transcription factors (RUNX1, CEBPA, NPM1) or epigenetic genes (DNMT3A, TET2, IDH1/2) or can be seen as a subclone in the process of clonal evolution triggered by a poly comb pathway of SF3B1, SRSF2 or a signaling mechanism involving JAK2, RAS, FLT3, PTPN11, or BCOR [59,61,114].

Recent studies have shown the importance of VAF to prognosticate the role of sub-clonal *TP53* mutation in AML. In the study *TP53* mutations were divided by their VAF into subgroups of >40%, 20–40%, and <20%. VAF of <20%, although considered sub-clonal, had a negative impact on overall survival (OS), complete remission rate and event free survival rates. However, a lower VAF was associated with fewer chromosomal losses and complex karyotype [118]. Similar results were validated with myelodysplasia and secondary AML patients, where in VAF <20% was associated with improved OS compared to VAF >40% which predicted complex karyotype and worse OS [119,120]. *TP53* can be associated with diploid cytogenetics but usually the VAF is lower compared to non-diploid cytogenetics.

Short et al. further validated these findings by demonstrating that AML with mutated *TP53* VAF of >40% was independently correlated with worse relapse free survival and OS [121].

Furthermore, Sallman et al. describe the striking relationship between an increase in VAF% and worsening karyotype complexity, which again correlated with worsening OS [119].

About 20% of patients with del 5q have *TP53* mutation clonal evolution, which is generally associated with resistance to lenalidomide and transformation to AML. As per the world health organization (W) 2016, monitoring for the appearance of clonal evolution or increase in the clone of *TP53* predicts disease progression [122,123,124,125].

The extent of *TP53* genome instability varies depending on whether it is a monoallelic *TP53* hit per single gene mutation or biallelic/multiple hits. Rate of transformation to AML from MDS and overall survival was worse with multiple *TP53* hits in comparison to monoallelic *TP53* hits- which was very similar to the *TP53* WT [126].

## 6. Current Standard Therapeutic Concepts

Therapy with standard chemotherapy in *TP53* mutated AML with loss of *TP53* allele displays a lack of chemotherapy induced apoptosis and subsequently a poor response rate (RR), disease-free survival (DFS) and OS while being independent of cytogenetic aberration [5] Decitabine (DAC) has been shown to enhance chemotherapy response in patients harboring *TP53* mutations, however, it has not been able to deliver a durable response or clear subclones of *TP53* mutations [127,128]. Nevertheless, a higher response was noted in AML patients with *TP53* mutations compared to WT *TP53* (100 vs. 41%) [127]. The underlying mechanism responsible for the sensitivity of *TP53* AML to DAC is currently uncertain. *TP53*-mutated AML patients receiving a monthly regimen of 5-day DAC had an overall response rate of 62% [12].

Short et al. assessed the efficacy of a 5-day DAC schedule compared to a 10-day regimen in a randomized phase II, open label, single center trial. There was no difference in outcome between the two DAC schedules for those with *TP53* mutated status. Intriguingly, response rate did not differ depending on baseline VAF. Four patients had a VAF ≥20% at the time of remission suggesting the presence of *TP53* mutation preleukemic clones rather than solely myeloblasts. The two patients that had the longest remission duration had no detectable *TP53* mutations present in the remission bone marrow sample [129]. It is widely understood that dominant clone *TP53* mutations have poor outcomes but recently sub-clones were noted to have equally poor outcomes [118].

*TP53* mutated cells are more sensitive to hypomethylating agents in comparison to cytotoxic agents, providing the impetus for a combination with bcl-2 inhibitor- Venetoclax (VEN) AML study. In patients harboring *TP53* mutations, the complete response (CR) + complete remission with incomplete blood count recovery (CRi) rate was 47% while the median OS was 7.2 months. Compared to historical standards of 28% CR rates, the combination displayed promising efficacy. VEN mediated apoptosis appeared *TP53* independent [130,131,132,133].

One of the primary and adaptive forms of resistance to VEN-based combination therapy is activated bi-allelic *TP53* perturbation. Hence, on disrupting *TP53* function within an AML cell line by using CRISPR/Cas9, DiNardo et al. were able to demonstrate an association between *TP53* loss and resistance to VEN, hypomethylating agent (HMA) and cytarabine (ARA-C) as a single agent or in combination [134]. Similarly, preclinical studies in leukemic cell lines have displayed an association between VEN resistance and a faulty apoptotic pathway involving *TP53* and BAX [135,136].

Allo-SCT with *TP53* mutation and complex cytogenetics portend inferior OS in patients with MDS. However, outcomes in patients with complex cytogenetics and WT *TP53* are not poor, solidifying the fact *TP53* mutation is a critical driver for poor overall survival [137].

## 7. Newer Molecularly Targeted Therapies

Relapses and primary resistance still occur in *TP53* mutated AML, necessitating the dire need for newer therapeutic options. The primary objective of newer therapies is to restore normal *TP53* function by either degrading or inactivating mutant p53 or reviving WT p53. (Table 1 and Table 2).

### 7.1. Mutant TP53 Inhibitor-APR-456

For the longest time *TP53* was considered undruggable. Eprenetapopt (APR-246), a PRIMA-1 analog, is a novel, first-in-class, small molecule therapeutic agent that binds covalently to cysteine residues in mutant p53 protein and induces a conformational shift to a WT-like structure.

This enables p53 binding to a specific DNA sequence leading to reactivation of pro-apoptotic function by enhancement of target gene transcription [138,139]. APR-246 has activity in cells without detectable *TP53* expression and in WT *TP53* cells by inducing heightened oxidative stress resulting in cell apoptosis [140] Due to a synergistic effect with azacytidine (AZA) in preclinical AML models, Eprenetapopt is being tested in a phase 1b/2 study in the treatment of naïve high risk myelodysplastic syndrome (HR-MDS) and oligoblastic (20–30% blasts) AML patients. Higher response rates were noted in patients with >10% p53 positivity by IHC and isolated *TP53* mutation. Median duration of response was 6.5 months and median time to response was 2 months. Of the 45 patients evaluated, overall response rate was 88% and 53% achieved CR [141].

A European phase 2 study with a similar cohort of HR-MDS and AML showed an overall response rate of 75% and CR of 56% [142]. *TP53* VAF clearance correlated with CR in both the studies. On the basis of these encouraging results, APR246 received fast track and orphan drug designation from FDA in April 2020. Results of the randomized phase 3 study of APR-246 and AZA compared to azacytidine alone (NCT03745716) is awaiting full report.

APR346 is being investigated in a phase I study in combination with AZA/VEN for newly diagnosed *TP53* mutated myeloid malignancies (NCT04214860) and being assessed in combination with azacitidine following allo-SCT as maintenance therapy in *TP53* mutated AML/MDS (NCT03931291).

APR-548, an orally bioavailable derivative of APR-246, is currently being developed to be used in a phase 1 study with AZA in *TP53* mutated MDS (NCT04638309). The synergy observed between APR-246 and AZA is hypothesized to be related to down regulation of FLT3 pathway [143].

### 7.2. MDM2 Inhibitors

p53 pathway dysfunction is highly prevalent in acute myeloid leukemia independent of *TP53* mutational status [31] MDM2 and MDM4 are negative regulators of *TP53* activity, targeting degradation via ubiquitination. In AML, MDM2 is often overexpressed, hence cells may have low functional *TP53* activity without *TP53* deletion/mutation. Nutlin-3a is a small molecular inhibitor of the MDM2/p53 interaction, thus this therapeutic provides increased p53 levels secondary to reduced p53 destruction, consequently upregulating apoptosis [144].

RG7388/idasanutlin is an orally bioavailable MDM2 inhibitor which was investigated in the placebo controlled, double blind phase III MIRROS study in combination with cytarabine in the management of relapsed refractory (r/r) AML. Unfortunately, the study had to terminate early as it did not meet primary end point of improved survival in comparison to ARA-C alone [145]. *TP53* stabilization, cell cycle arrest and apoptosis were produced in a dose dependent fashion [146]. *TP53* mutational status alone did not correlate with CR, although increased expression of MDM2 among HSPC and leukemia blasts was consistent with CR [147,148].

Idasanutlin is currently in clinical trials in combination with Venetoclax in r/r AML (NCT02670044 and NCT04029688). The preliminary results were presented at the 2019 American Society of Hematology annual meeting, and the CR/Cri was reported in 11 of 49 patients [149]. It is in clinical trials for newly diagnosed AML in combination with cytarabine and daunorubicin based induction regimen (NCT03850535).

AMG 232 is a structurally distinct MDM2 inhibitor and interacts with the glycine self-region of the *TP53* binding pocket on the surface of MDM2 [150]. AMG 232 is being investigated in r/r AML treatment.

Of the 30 patients evaluated, four achieved a morphological leukemia-free state. Unfortunately, no response was noted in patients with *TP53* mutations.

AML possesses high expression of MDMX. Current MDMD2 inhibitors provide a limited effect on other members of the MDM family, including MDMX, which decreases their utility in AML treatment [151]. ALRN-6924 is a stapled peptide with dual inhibition of MDM2/MDM4. Phase I clinical trials utilizing MDM2/MDMX inhibitors in r/r AML and MDS are underway [151,152,153].

### 7.3. Immunotherapy and Other Agents

Cell surface CD47 interacts with its corresponding receptor on macrophages to inhibit the phagocytosis of normal, healthy cells. CD47 seems to be over expressed in myeloid malignancies, and overexpression of CD47 in myeloid leukemia increases its pathogenicity by allowing for tumor evasion of macrophages. Inhibition of CD47 induces engulfment of leukemic cells [154]. Magrolimab, a CD47 inhibitor, was investigated in a phase 1b study either as single agent in r/r AML or in combination with azacitidine in newly diagnosed AML. Combined Magrolimab and AZA was assessed in the treatment *TP53* mutated AML patients (*n* = 9) resulting in a CR/Cri of 78% and 44%, respectively and negative MRD in 57% of the responders, with a median follow up of 6.9 months. Median duration and survival were not reached [155].

Arsenic trioxide has been shown to inactivate *TP53* via 26S proteasome and upregulate WT *TP53* functions, thereby inactivating proliferating leukemia cells and promoting apoptosis [156,157]. Furthermore, atorvastatin (statin), a cholesterol lowering drug, was recently investigated as an inducer of the degradation of misfolded or conformational mutant *TP53* with minimal effects on WT p53 and DNA contact mutants [158].

## 8. Conclusions

There is growing evidence of age-related mutations and CH in the HSC and the influence of *TP53* on normal HSC self-renewal. The impact of *TP53* mutations on CH is indisputable. Improved characterization of the role of *TP53* in normal hematopoiesis will lead to a more complete understanding of *TP53* mutations in the propagation of CH and leukemogenesis. Pathways leading to *TP53* dysregulation represent a common element in non-*TP53* mutated AML, hence leveraging WT *TP53* in unmutated AML serves as a corner stone in the design of anti-AML therapeutic strategies.

Recently, it has become understood that unaltered *TP53* and mutated *TP53* within tumor cells can be therapeutically targeted, which has led to the use of novel therapies like, APR-456 which has reported to restore transcriptional activity in mutant p53 or unfolded WT p53, leading to apoptosis or the promising result of anti CD47monoclonal antibody magrolimab in TP53 mutated AML. 

This approach is of great precedence, as *TP53* mutations in AML are distinctive, although rare, and confer dismal responses to chemotherapeutic agents with very poor outcomes.

Hence, research focused on the exploitation of *TP53* pathway activators may yield immense contributions to the management of AML, a pathology associated with a very high risk of therapy failure.

## Figures and Tables

**Table 1:** Clinical trials to restore wild type function of p53.

Target	Clinical Trial Number	Antineoplastic Combination	Compound	Phase (Status)
MDM2	NCT01773408	ARA-C	Idasanutlin/Cometinib	I/IB (Completed)
	NCT02098967	Alone or AZA	Milademetan	I (Completed)
	NCT02545283	ARA-C	AMG-232	III (Recruiting)
	NCT03671564	DAC	HDM201	I (Recruiting)
	NCT02319369	Alone or AZA	DS-3032b/Milademetan	I (Recruiting)
	NCT03634228		Ara-Ca	I/II (Recruiting)
	NCT03041688	DAC	AMG-232	IB (Recruiting)
	NCT02143635		HDM201	I (Recruiting)
MDM2 & BCL2	NCT02670044		Dasanutlin/VEN	IB/II (Recruiting)
MDM2 & MDMX	NCT02909972	Alone or AZA	ALRN-6924	I (Recruiting)

**Table 2:** Clinical trials to degrade mutant p53.

Target	Clinical Trial Number	Antineoplastic Combination	Compound	Phase (Status)
Mutant *TP53*	NCT03072043	AZA	APR-246/PRIMA-1Met	IB/II (Recruiting)
Several targets	NCT03381781	DAC	Arsenic Trioxide	II (Recruiting)
HMG-CoAreductase	NCT03560882		Atorvastatin/Lipitor	I (Recruiting)
